# *LOXL1* genetic polymorphisms are associated with exfoliation glaucoma in the Japanese population

**Published:** 2008-06-05

**Authors:** Kazuhiko Mori, Kojiro Imai, Akira Matsuda, Yoko Ikeda, Shigeta Naruse, Hisako Hitora-Takeshita, Masakazu Nakano, Takazumi Taniguchi, Natsue Omi, Kei Tashiro, Shigeru Kinoshita

**Affiliations:** 1 Department of Ophthalmology, Kyoto Prefectural University of Medicine, Kyoto, Japan; 2 Genomic Medical Sciences, Kyoto Prefectural University of Medicine, Kyoto, Japan

## Abstract

**Purpose:**

We performed genetic association studies using a native Japanese population to examine the reproducibility of results of *lysyl oxidase-like 1* (*LOXL1*) genetic association studies for exfoliation glaucoma (XFG) beyond the differences of ethnicity. We also quantified *LOXL1* mRNA expression in the human lens capsule to examine the possible correlation between *LOXL1* expression and XFG pathogenesis.

**Methods:**

We performed a case-control study using 95 Japanese XFG patients and 190 controls. Real-time polymerase chain reaction (PCR) analysis was performed using lens capsules obtained during surgery.

**Results:**

The TT genotype in the single nucleotide polymorphism (SNP) rs1048661 and the GG genotype in the SNP rs3825942 in exon 1 of *LOXL1* were significantly associated with an increased risk of XFG under recessive models (χ^2^ test, p=5.34×10^−34^ and p=2.1×10^−8^, respectively). Quantification of *LOXL1* mRNA expression demonstrated no significant difference between XFG and senile cataract samples.

**Conclusions:**

Although the functional effects of the *LOXL1* SNP appear to be qualitative rather than quantitative, the amino acid substitution (R141L) caused by SNP rs1048661 is not a simple decisive factor for XFG due to the inverted allele frequency between Japanese XFG and Caucasian XFG patients. Further genetic and functional studies are essential for clarifying XFG pathogenesis.

## Introduction

Exfoliation glaucoma (XFG) is an age-related disorder associated with exfoliation syndrome (XFS), manifested by abnormal fibrillar deposits on the lens and iris epithelium [1]. Recently, a genome wide association study performed for the Caucasian population revealed a strong association between the genotype of single genetic polymorphisms (SNPs) in the *lysyl oxidase-like 1* (*LOXL1*) gene and the occurrence of XFS/XFG [2]. It was reported that the rate of XFG occurrence significantly differed from one ethnic population to another [3], therefore it is logically important to perform a case-control study using another ethnic population such as the Japanese. In this study, we found a strong genetic association between the occurrence of XFG and the *LOXL1* single nucleotide polymorphism (SNP) genotype. One of the nonsynonymous SNP (rs1048661) showed a very strong association with XFG. However, the risk allele was inverse compared to the Caucasian study. To gain further insight into the role of *LOXL1* for XFG, it is important to compare the expression levels of *LOXL1* mRNA in XFG eyes and non-glaucomatous eyes. We obtained anterior lens capsules during combined glaucoma-cataract surgery or during cataract surgery alone from XFG patients and non-glaucomatous senile cataract patients, respectively. We then performed a quantitative analysis of *LOXL1* mRNA expression using these anterior lens capsules.

## Methods

### Subjects

All XFG patients were diagnosed by slit-lamp examination for the existence of exfoliation material on the anterior lens capsule with maximal dilation of the pupils and with glaucomatous optic neuropathy as well as visual field defect. Peripheral blood was obtained from 95 XFG patients 47–93 years of age (mean age: 75.7±8.1 years). The controls were 190 randomly-selected, population-based individuals 54–83 years of age (mean age: 65.0±6.8 years) with no glaucomatous changes or existence of exfoliation materials (Table 1). All of the XFG patients and normal volunteers were recruited at Kyoto Prefectural University Hospital (Kyoto, Japan) and examined by glaucoma specialists using slit-lamp microscopy and an automated visual field analyzer. All study subjects were ethnically Japanese. According to the rules of the process committee at Kyoto Prefectural University of Medicine, written informed consent was obtained from all participants before participation in this genetic association study. The study was conducted in accordance with the tenets of the Declaration of Helsinki.

**Table 1:** Clinical characters of the exfoliation glaucoma patients and control.

	**XFG**	**Control**
Total number of subjects	95	190
Mean age (range)	75.7 (47–93 years)	65.0 (54–83 years)

Ninety-five exfoliation glaucoma patients 47 to 93 years of age (mean age: 75.7 years), and 190 randomly-selected population-based individuals 54 to 83 years of age (mean age: 65.0 years) with no glaucomatous changes or existence of exfoliation materials were involved in this study.

### Genotyping

We genotyped two nonsynonymous single nucleotide polymorphisms (SNPs; rs1048661 and rs3825942) in the *LOXL1* gene region according to the previous report [2]. We genotyped the SNPs with both direct sequencing and the TaqMan genotyping assay (Applied Biosystems, Foster City, CA). We used a set of primers (5′-GAT CCA GTG GGA GAA CAA CG-3′ and 5′-GGT ACT CGG GCA GCT CTT C-3′) for direct sequencing. Genotyping was performed using on a 3130xl Sequence Detection System or with 7500 Realtime-time polymerase chain reaction (PCR) system (Applied Biosystems). The TaqMan genotyping assay was performed according to the manufacturer’s protocol. All the genotyping procedures were approved by the ethics committee of Kyoto Prefectural University of Medicine.

### Statistical analysis

The frequencies of the genotypes were compared between XFG patients and controls in the recessive model. In this model, frequencies of the homozygous genotype for major alleles were compared using a 2x2 contingency table. Here, the association was evaluated using the χ^2^ test for the contingency table. A p-value of less than 0.01 was considered to be statistically significant. Odds ratios (OR) and 95% confidence intervals (95% CI) were also calculated.

### Real-time polymerase chain reaction analysis

Anterior capsules that were obtained during glaucoma/cataract or cataract surgery with written informed consent were immediately stored with RNAlater reagent (Ambion, Austin, TX) to protect the RNA. All procedures were approved by the ethics committee of Kyoto Prefectural University of Medicine. Total RNA was isolated with the Micro RNA extraction kit (Qiagen Japan, Tokyo, Japan) from the anterior capsules, and then cDNA was prepared as described previously [4]. The anterior capsules were obtained from 10 XFG patients undergoing combined (cataract+glaucoma) surgery (mean age 74.9±8.0 years; male:female=6:4) and 10 non-glaucomatous, senile cataract patients (mean age: 76.5±10.6 years; male:female=4:6). To avoid contamination of blood, anterior lens capsulotomy was performed before glaucoma surgery in cases of combined procedures. We used TaqMan real-time PCR probes and primers specific for human *LOXL1* (Hs00173746_m1), and 18S rRNA from Applied Biosystems (Assay-on-Demand gene expression products). Real-time PCR analysis was performed on a 7500 real-time PCR system. The relative expression of *LOXL1* mRNA in the anterior lens capsule was quantified by the standard curve method using 18S rRNA expression in the same cDNA as the control.

## Results

### Case-control association study in the *LOXL1* gene region

We genotyped 95 XFG cases and 190 control subjects by direct sequencing methods. The rs1048661 TT genotype and the rs3825942 GG genotype in the first exon of *LOXL1* were significantly associated with an increased risk of XFG under recessive models (χ^2^ test, raw p value=5.34x10^−34^, OR=321.3, 95% CI=43.5–2373.2 and p=2.1x10^−8^, OR=38.3, 95% CI=5.2–281.6, respectively; Table 2). We further tested reproducibility of the genotyping by TaqMan genotyping assay and confirmed the genotyping results because the results of the rs1048661 genotyping was not concordant with the Hardy–Weinberg equilibrium (HWE). The results obtained by the two methods were identical.

**Table 2:** Case-control study of two nonsynonymous single nucleotide polymorphisms in *LOXL1*.

rs1048661 (R141L)*			rs3825942 (G153D)**		
**Genotype**	**Control**	**XFG**	**Genotype**	**Control**	**XFG**
GG	33	0	GG	135	94
GT	114	1	GA	53	1
TT	43	94	AA	2	0

The rs1048661 TT genotype and the rs3825942 GG genotype were significantly associated with an increased risk of XFG under recessive models (χ^2^ test). The single asterisk indicates TT versus GT+GG, P value=5.34x10–34, OR=321.3, and 95% CI=43.5–2373.2, and the double asterisk indicates GG versus GA+AA, p=2.1x10–8, OR=38.3, and 95% CI=5.2–281.6.

### Haplotype analysis of *LOXL1* single nucleotide polymorphisms

First, we checked the state of linkage disequilibrium (LD) between rs1048661 and rs3825942 and found that those two SNPs are in a state of strong LD (Table 3). Next, we examined the distribution of two-locus haplotypes in the XFG and control samples (Table 4) using Haploview [5] and PENHAPLO [6] software. Among the two-locus haplotypes of SNPs in exon 1 (rs1048661 G/T and rs3825942 G/A), the TG haplotype showed an increased risk for XFG (TG/TG versus others; p=6.87x10^−7^, OR=312.162) in recessive models. We checked HWE among control subjects using this two-locus haplotype and found that the two-locus haplotype was concordant with HWE (p=0.0125, χ^2^ test).

**Table 3:** Linkage disequilibrium state of rs1048661 and rs3825942.

**SNP**	**D'**	**r2**
Control+XFG	1	0.243
Control only	1	0.196
XFG only	1	1

We calculated the state of linkage disequilibrium between rs1048661 and rs3825942 among the control population, XFG population, and total population. Linkage disequilibrium coefficients were expressed as D' or r2.

**Table 4:** Structures and frequency of two locus haplotype.

**Haplotype**	**Frequency**	
	**Case**	**Control**
TG	0.9947	0.5263
GG	0	0.3237
GA	0.0053	0.15

We examined the frequency of two-locus haplotypes in the XFG and control samples using Haploview and PENHAPLO software. The TG haplotype means rs1048661-T and rs3825942-G, the GG haplotype means rs1048661-G and rs3825942-G, and the GA haplotype means rs1048661-G and rs3825942-A.

### *LOXL1* mRNA quantification using anterior lens capsules

**Figure 1:**
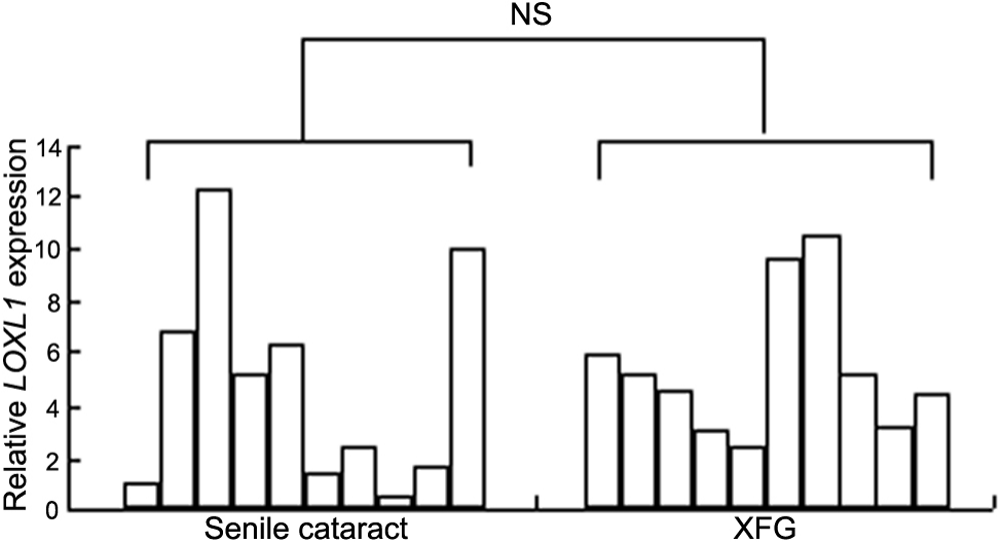
Real-time polymerase chain reaction analysis of *LOXL1* mRNA expression in anterior lens capsules. Total RNA was extracted from the anterior lens capsule of XFG/senile cataracts. Real-time PCR analysis was performed with expression assay probes. The amount of relative expression was normalized to that of 18Sr RNA. (N.S.: Not statistically significant, p=0.529; Mann–Whitney’s U-test).

cDNA was synthesized from total RNA isolated from the anterior lens capsules of patients undergoing cataract surgery. We analyzed both XFG (n=10) and senile cataract (n=10) specimens. Figure 1 is a representative result of two independent results of experiments run in duplicate. There was no statistically significant difference between the expression levels of *LOXL1* mRNA in the lens epithelium obtained from XFG patients and senile cataract patients. (Figure 1, p=0.529 by the Mann–Whitney U*-*test)

## Discussion

Similar to previous studies among Caucasian [2,7,8], we found a strong genetic association between the *LOXL1* SNP and XFG patients. The lysyl oxidase protein family has multiple functions including specific oxidative deamination of lysine residues and the cross-linking of elastin [9]. Phenotypic analysis of *LOXL1* knockout mice [10] showed that the LOXL1 protein has an essential role for the homeostasis of elastic fibers, which also contribute to the trabecular meshwork structures [11]. Therefore, it is functionally reasonable to assume that *LOXL1* is one of the causative genes for XFG.

For the rs3825942 genotype, our results are consistent with those of Thorleifsson et al. [2]. The allele frequency of rs3825942 G is consistently higher among Caucasian and Japanese XFG patients so it is reasonable to conclude that rs3825942 A is a protective allele against the occurrence of XFG.

On the other hand, the risk allele for XFG occurrence is rs1048661-“T” among the Japanese population and rs1048661-“G” among the Caucasian population. We double-checked these genotype results (rs3825942 and rs1048661) by both direct sequencing and the TaqMan genotyping assay because the genotype results of rs1048661 within control subjects were not concordant with HWE. The results obtained from two different methods were in complete agreement. Therefore non-concordance with HWE is not due to genotyping errors. We further checked the two-locus haplotype and found that the haplotype was concordant with HWE. Since the two-locus, rs1048661 and rs3825942, was in the state of linkage disequilibrium (D’=1.0), it was appropriate to check the HWE by haplotype.

Inverted genotypes of XFG patients between the Japanese and Caucasian population suggests the following possibilities: (1) The 141st amino acid substitution (R141L) does not have a dominant role for the pathophysiology of XFG, (2) the heterozygote for the rs1048661 G/T genotype may have a protective role against XFG occurrence, or (3) there might be another causative polymorphism in a state of linkage disequilibrium with rs1048661 G/T. If the last possibility is the case, there might be a historical recombination between the SNP rs1048661 and the causative polymorphism.

As a next step, we quantified relative *LOXL1* mRNA expression in anterior lens capsules and found that there was no significant difference between XFG and senile cataract controls. These results are different from the previous study by Thorleifsson et al. [2], which showed a significantly higher *LOXL1* mRNA expression with the TT genotype (the protective genotype against XFG in the Caucasian population) than those with the TG or GG genotype using adipose tissues. We hypothesize that the difference might be due to the mixed *LOXL1* SNP genotype of our senile cataract surgery samples, which are not able to clarify genotype due to ethical procedure reasons. However, our *LOXL1* mRNA expression analysis reflects a direct pathological site within the ocular tissue and better represents *LOXL1* expression status in the affected eyes than that of adipose tissue [2]. Therefore, we deduced that it is likely that the quantitative difference of *LOXL1* mRNA is not a direct pathogenetic cause of XFG.

To further clarify the pathophysiological role of *LOXL1* for XFG, we are now performing extensive SNP discovery around *LOXL1* to find out other possible causative polymorphisms. In addition, functional analysis of LOXL1-fibulin5 protein interaction [12] using two types of recombinant LOXL1 precursor protein (141R and 141L) is ongoing. Since LOXL1-fibulin5 interaction is essential for mature cross-linked elastin formation [10,12], we are focused on determining the role of the two variants of *LOXL1* and its mixture to gain further insight into our genetic association results showing that the rs1048661 G/T heterozygote is protective against XFG. It is also important to investigate the protective role of LOXL1 protein variants that possess the 153D amino acid, which is indicated by multiple genetic association studies including our own [2,7,8] as well as to analyze the possible role of the pairs of 141/153 amino acid variants in consideration of our haplotype analysis result.
